# Kita-Kyushu Lung Cancer Antigen-1 (KK-LC-1): A Promising Cancer Testis Antigen

**DOI:** 10.14336/AD.2021.1207

**Published:** 2022-07-11

**Authors:** Rui Bai, Cheng Yuan

**Affiliations:** ^1^Department of Radiation and Medical Oncology, Zhongnan Hospital of Wuhan University, Wuhan, Hubei, China.; ^2^Department of Gynecological Oncology, Zhongnan Hospital, Wuhan University, Wuhan, China.

**Keywords:** KK-LC-1, Cancer testis antigen, Immunotherapy, Carcinogenesis, Immune target

## Abstract

Cancer has always been a huge problem in the field of human health, and its early diagnosis and treatment are the key to solving this problem. Cancer testis antigens (CTAs) are a family of multifunctional proteins that are specifically expressed in male spermatozoa and tumor cells but not in healthy somatic cells. Studies have found that CTAs are involved in the occurrence and development of tumors, and some CTAs trigger immunogenicity, which suggests a possibility of tumor immunotherapy. The differential expression and function of CTAs in normal tissues and tumor cells can promote the screening of tumor markers and the development of new immunotherapies. This article introduces the expression of Kita-Kyushu lung cancer antigen-1 (KK-LC-1), a new member of the CTA family, in different types of tumors and its role in immunotherapy.

## 1. Introduction

Cancer testis antigens (CTAs) are a class of proteins encoded by 276 genes that are only expressed in the testes, not in any other normal tissues [1-3]. Approximately half of the CTA genes are encoded on the X chromosome, so they are called CT-X genes [4,5]. These CTAs belong to the typical testicular restriction type and are more immunogenic than non-CT-X genes located on autosomes [6].

CTAs were first reported in 1977 and have been found to be abnormally expressed in a variety of cancers, including melanoma, head and neck cancer, lung cancer, liver cancer, gastric cancer, ovarian cancer and breast cancer [7-13]. CTAs are not only closely related to the stemness of tumor cells, tumorigenicity, mobility, metastasis and the drug resistance of cancer cells [3,14-25], but they also show high tumor specificity and sensitivity [26-30]. Because of these characteristics, CTAs are considered to be tumor-specific markers that can be used in cancer diagnosis and prognostication, and possibly as targets for cancer treatment [31-33].

The use of autologous typing is a turning point in the search for tumor antigens. Autologous typing is a method that uses autoantibodies and T cells of cancer patients to identify tumor cells and normal cells to determine whether the patient has a tumor-specific T cell response and antibodies [34]. Using this method, a series of tumor antigens were found, including viral oncoproteins, mutant proteins, fusion proteins, overexpressed proteins, differentiation proteins and cancer testis antigens.

The first CTA was cloned by van der Bruggen and others in 1991 [35], and then BAGE [36] and GAGE [37] were discovered one after another. In 1995, Sahin U’s team improved the identification of CTA through serological analysis of a recombinant cDNA expression library (SEREX) and used autologous patient serum to screen a phage display library derived from tumor cDNA [38]. This technology contributes to the discovery of a variety of tumor antigens, including NY-ESO-1, the most successful tumor immunotherapy target thus far [39]. Subsequently, to reflect the expression specificity of such tumor-associated antigens, CHEN et al. introduced the term "cancer/testis antigen", namely, CTA [40].

At present, the CTA database (www.cta.lncc.br/index.php) includes more than 200 genes, and the number is still increasing. These tumor antigens mainly include mutated genes and overexpressed genes that are ubiquitous in tissues, as well as genes that are not expressed in normal adult cells but are expressed in cancer cells. At present, more than 730 CTAs have been identified, but it is still uncertain whether some CTAs identified by expression data are immunogenic [41-43]. In this article, we will introduce the characteristics and therapeutic prospects of Kita-Kyushu lung cancer antigen-1 (KK-LC-1), a newly discovered member of the CTA family.

KK-LC-1, whose full name is Kita-Kyushu lung cancer antigen-1, also known as CT83 or cxorf61, was discovered by Takashi Fukuyama's team in 2006[44]. Takashi Fukuyama derived lung adenocarcinoma cell lines from the tumors of lung cancer patients, stimulated the regional lymph node cells of the patients, induced cytotoxic T lymphocyte (CTL) clones, and then identified new antigen coding genes by screening a cDNA library from allogeneic lung cancer cell lines.

KK-LC-1 is located on chromosome Xq22 and consists of 556bp. It is not expressed in normal tissues except the testis but is highly expressed in lung cancer, gastric cancer and breast cancer. At present, it has been reported that KK-LC-1 plays a role in the immune response as a new antigen, but the structure and function of this gene are not fully understood. How this gene plays a role in diseases remains to be further studied.

## 2. Expression and significance of KK-LC-1 in different tumors

KK-LC-1 is abnormally expressed in different types of cancers, including lung cancer, gastric cancer, breast cancer and liver cancer [44-47], but the biological function and potential mechanism of KK-LC-1 in cancer are still unclear.

### 2.1 Gastric cancer

In 2015, Masahiko Watanabe's team found that KK-LC-1 was highly expressed in gastric cancer, and the expression was higher than that of other CTAs, such as MAGE-A1, MAGE-A3 and NY-ESO-1 [48]. In 2017, Masahiko Watanabe's team found that the expression of KK-LC-1 in stage I gastric cancer was very high, suggesting that KK-LC-1 can be used as a potential marker for the early diagnosis and treatment of gastric cancer [49]. In the same year, Noritada Kobayashi's team found that KK-LC-1 was highly expressed in gastric cancer caused by *Helicobacter pylori* infection, suggesting that *Helicobacter pylori* infection may induce the expression of specific CTAs [45]. In 2020, Yoshihito Takahashi's team found that the expression of KK-LC-1 in gastric cancer was related to *Helicobacter pylori* infection and atrophy [50]. A combination of improved ABCD methods (serological detection of *Helicobacter pylori* (HP) antibodies and the pepsinogen (PG) method for risk stratification of gastric cancer) and KK-LC-1 detection may improve the accuracy of diagnosis. To better detect the expression of KK-LC-1, in 2019, Noritada Kobayashi's team synthesized a new antibody, Kmab34B3, which can successfully detect KK-LC-1 in gastric cancer cells and tissues [51].

### 2.2 Liver cancer

By examining 60 pairs of liver cancer and paracancerous tissues, the researchers determined that KK-LC-1 is highly expressed in liver cancer and is closely related to the prognosis of liver cancer [47]. A series of phenotypic experiments were carried out by knocking down or overexpressing KK-LC-1. KK-LC-1 can promote the proliferation, invasion and migration of hepatocellular carcinoma cells. An animal model further verified the tumor-promoting effect of KK-LC-1 in vivo. In terms of the mechanism, the researchers found that KK-LC-1 can regulate the expression of the Notch1 intracellular domain (NICD1) and Notch1 effector Hes1. In vivo experiments also verified the correlation between KK-LC-1 and NICD1. Further exploration found that KK-LC-1, through interaction with presenilin-1, activates the Notch1 signaling pathway and then it plays a role in promoting the growth, migration and invasion of liver cancer, thus triggering the tumorigenesis of liver cancer. Presenilin-1 is the catalytic subunit of endosynthesis that can catalyze the Notch1 gene. These results suggest that high levels of KK-LC-1 may be an independent predictor of poor survival in liver cancer patients [47].

### 2.3 Breast cancer

In 2018, Masahiko Watanabe's team analyzed the surgical specimens of 51 patients with breast cancer and found that KK-LC-1 could be detected in triple-negative breast cancer cases and all tumors without estrogen receptor expression, and its expression level in tumor tissues was significantly higher than that in paracancerous tissues. KK-LC-1 can be used as a marker for the clinical diagnosis and immunotherapy of breast cancer [46].

### 2.4 Lung cancer

In 2018, a team from a university in China constructed a CTA map of stage III lung cancers requiring surgery through a comprehensive analysis of 10 kinds of CTAs of NSCLC [52]. The purpose of this study was to find the most appropriate CTA indicators to assist in decision-making. At the same time, the CTA maps also had therapeutic potential for TCR-T cell treatment.

Takeshi Hanagiri's team found that the positive rate of KK-LC-1 was 30.9% by examining clinical samples of surgical resection of non-small-cell lung cancer (NSCLC). The decreased expression of class I molecules indicates a poor prognosis among patients with positive CTAs and is an important obstacle to tumor antigen immunotherapy. This provides a new idea for the immunotherapy of CTAs, and the future synergistic therapy of CTAs and HLA may be a new breakthrough [53].

## 3. Application of targeted KK-LC-1 in tumor immunotherapy

**Figure 1.**
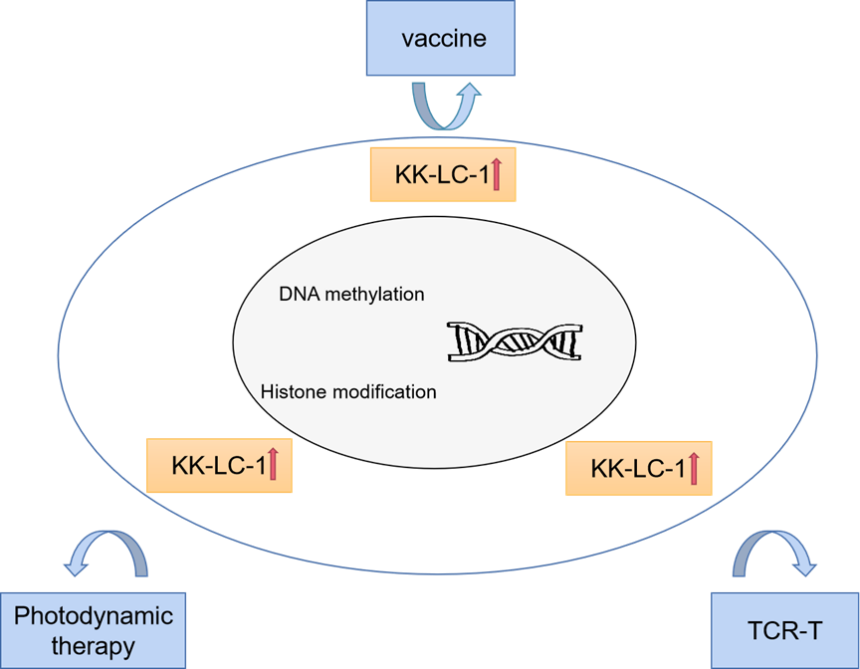
**Targeting KK-LC-1 for effective cancer immunotherapy**. The strategies for effective cancer immunotherapy focused on KK-LC-1 include vaccines, photodynamic therapy, and TCR-T cells. The mechanism of CTA reactivation may be DNA methylation and histone modification.

In view of the high immunogenicity and tumor specificity of CTAs, carcinogenic CTAs are sensitive targets for cancer immunotherapy [54-64]. In recent decades, several targeted immunotherapies for carcinogenic CTAs have been developed, and these immunotherapies have been tested in preclinical and early clinical environments. At present, most clinical studies have focused on the treatment of melanoma-associated antigen A (MAGEA) and New York esophageal squamous cell carcinoma-1 (NY-ESO-1), including cancer vaccines targeting CTAs to prevent tumor occurrence and development, and monoclonal antibodies against CTAs and CAR-T designed based on CTAs [65-73]. Due to the late discovery of KK-LC-1, the reported treatments include vaccines, photodynamic therapy combined with new photosensitizers and TCR-T therapy (Fig. 1).

### 3.1 Vaccine

The vaccine designed for DNA of MAGE-A in 2018 has shown anticancer properties in a number of clinical trials[74]. A clinical trial published in Nature in 2020 showed that liposome RNA (RNA-LPX) vaccines based on MAGEA, and two other kinds of CTAs could induce strong cellular immunity [75]. Because KK-LC-1 is also antigen-specific, we suspect that it is possible to synthesize a vaccine against KK-LC-1. A new paper in 2021 reported the design of a multiepitope vaccine against MAGEA3, MAGEA4, NY-ESO-1 and KK-LC-1 using reverse vaccinology for the first time [76]. The main idea is to draw the epitope map according to the CTA sequence, estimate the population coverage (PC) of the CD4+ and CD8+ epitopes, and then connect the candidate linear B cell (BL), CD4+ and CD8+ epitopes into a multiepitope structure (Mvax) using the flagellin domain as an adjuvant. After successful construction, the tertiary structure of Mvax was modeled and verified, and the antigenicity and cross-reactivity of Mvax was tested. It is critical that all epitopes contained in the vaccine dock with their human leukocyte antigen (HLA) conjugates to better act on the antigenic fragments presented by HLA. Finally, the researchers successfully designed a multiepitope vaccine targeting CTAs in NSCLC. Computer evaluation showed that Mvax has antigenicity, immunogenicity, stability and safety and is expected to be used in in vitro and in vivo studies.

### 3.2 Photodynamic therapy

Photodynamic therapy (PDT) is a noninvasive and highly selective tumor therapy, but its therapeutic effect has been limited by skin phototoxicity for a long time. Therefore, to overcome this disadvantage, it is best to selectively deliver photosensitizers to tumor cells with the help of specific antibodies against tumor-associated antigens. Some researchers developed and identified a new mouse monoclonal antibody (CT83 MAb7G4) against human CT83, which can effectively combine with the new photosensitizer gallium (III)5,10,15-tris (ethoxycarbonyl) corrole (1-Ga) to form the antibody-photosensitizer complex 7G4-1-Ga [77]. Enzyme-linked immunosorbent assays (ELISA), flow cytometry and cytotoxicity assays showed that 7G4-1-GA had high specificity for CT83. In addition, 7G4-1-Ga has a stronger cytotoxic effect on human tumor cells expressing CT83 than 1-Ga in vitro. These results suggest that a combination of anti-CT83 monoclonal antibody and antibody-conjugated photosensitizer 1-GA may have good application prospects in tumors with high expression of CT83.

### 3.3 TCR-T

TCR-T is used to extract the α and β chain genes encoding TCR from effector T cells induced by tumor antigens. It is introduced to mature T cells by genetic engineering technology, and then transfused back into patients who lack tumor antigen specific response T cells, so that recipient T cells express antigen specific TCR and exert the function of effector T cells. At present, the cancer testis antigen NY-ESO-1 is mostly being studied in clinical research [78]. The expression of cancer testis antigen is very low in normal tissues, and the probability of off-target effects is low. It is an ideal target antigen for adoptive cellular immunotherapy [79]. NY-ESO-1 TCR-T therapy was used in 20 patients with multiple myeloma, the clinical response rate was 80%, and TCR was continuously expressed in vivo for more than two years [21]. Eighteen patients with metastatic synovial sarcoma and 20 patients with melanoma were treated with TCR-T, and the clinical response rates were 61% and 55%, respectively [3]. Given that KK-LC-1 and NY-ESO-1 have many similar features, we suspect that TCR-T can also be designed for KK-LC-1.

In 2019, Bridget Marcinkowski et al. designed TCR-T presented by HLA-A*01:01, targeting KK-LC-152-60[80]. The researchers tested whether T cells transduced with KK-LC-1 TCR (KK-LC-1TCR-Ts) could recognize tumor cell lines expressing KK-LC-1 and HLA-A*01:01 in vitro. In the overnight coculture test, TCR-T cells recognized cell lines expressing target antigens and HLA-limiting elements and released interferon-γ. To evaluate whether KK-LC-1 TCR-T cells can mediate the antitumor response in vivo, researchers constructed a mouse xenograft model. The results showed that the model with low expression of KK-LC-1 on the surface of tumor cells was prone to relapse, and the model with high expression of KK-LC-1 could make the tumor regress after a single intravenous injection of KK-LC-1 TCR-Ts. These results suggest that KK-LC-1 TCR-Ts can prevent tumor progression both *in vitro* and *in vivo*.

## 4. Potential regulatory mechanism of KK-LC-1

CTAs are immunogenic proteins, so they can trigger cellular immunity and humoral immunity. Given that their expression in adult somatic tissues is greatly restricted and has immunogenic potential, CTAs are considered good candidate targets for cancer immunotherapy. CTA-based treatments include antibodies, vaccines and anti-CTA chimeric antigen receptor-modified T cells (CAR-Ts). Although several CTA-targeted therapies have achieved encouraging results in preclinical and early clinical trials, the anticancer effect of CTA-targeted immunotherapy is not ideal, so these immunotherapies have not yet been used in the clinic as first-line anticancer drugs [81-83].

Although immunotherapy for CTAs is theoretically feasible, it is difficult to implement immunotherapy due to the low and local expression of CTAs in tumors [84,85]. Therefore, we urgently need to explore the transcriptional regulation mechanism of CTAs in tumor cells to promote the expression of CTAs. It has been reported that the reactivation of CTAs is primarily due to changes in DNA methylation in the genome [5,20,86,87]. Bisulfite sequencing showed that compared with the corresponding normal tissues, the promoter region of CTAs in many tumors was hypomethylated, resulting in increased protein expression [88,89]. There are abundant CpG islands in the promoter region of the CTA genes, which is an important reason why these genes are sensitive to methylation regulation.

Weber et al. proposed for the first time that the treatment of tumor cells with dexitabine, an inhibitor of DNA methyltransferase, can reactivate the expression of MAGEA1 [90]. This suggests that demethylation of genomic DNA can activate CTA expression. Later, it was gradually found that DNA demethylation could reactivate the expression of NY-ESO-1, KK-LC-1 and other CTAs genes [66,91-96]. Researchers found that the DNA methylation level is negatively correlated with the expression level of KK-LC-1. Experiments with 5-aza-2'-deoxycytidine (5-aza-dC), methylation-specific PCR (MSP), and bisulfite sequencing PCR (BSP) also confirmed this conclusion.

In addition to DNA demethylation, which is considered to be the main factor in the activation of CTAs, histone acetylation also contributes to the transcriptional activation of CTAs and enhances the activation of tumor testis antigen genes by DNMTi [97]. Inhibition of HDAC alone could not activate the transcription of CTAs, but its combination with a DNA methyltransferase inhibitor could significantly enhance the transcription of CTAs. These strategies can effectively promote the expression of CTAs to enhance their immunogenicity, thus improving the response to T cell-based therapy [98].

Although epigenetic mechanisms play an important role in regulating the expression of tumor testis antigen genes, there is growing evidence that other nonepigenetic mechanisms also play a key role. At present, these mechanisms are not well understood, and the mechanisms currently recognized include sequence-specific transcription factors, signal transduction and activated tyrosine kinases. For example, two Ets binding sites are involved in the transcriptional activation of the MAGE1 gene [99-101], tyrosine kinase affects the methylation and expression of the MAGE gene promoter [102-107], cAMP increases the expression of MAGE-A11 in prostate cells [108], SP1 increases the expression of NY-ESO-1 in lung cancer cells [109], and there is a loop regulation between p53 and tumor testis antigen [110]. BORIS itself is a member of the CTA family [111-114], which is reactivated by hypomethylation in cancer. It can bind to SET1A H3K4, a methyltransferase associated with histone transcriptional activity modification, and then regulate the expression of other members of the CTA family [115]. The mechanism of epigenetic reactivation of KK-LC-1 expression remains to be further studied.

## 5. Discussion

At present, the exploration of KK-LC-1 is based on clinical research. To further understand the mechanism of KK-LC-1, more basic research needs to be carried out. The starting point can be carried out from the following two aspects:

There are antibodies available against NY-ESO-1, PRAME, and CT45. In 2019, Seth M. Pollack’s team used the antibody LV305 against NY-ESO-1 for the first time in the clinic, and the results showed good safety and antigen-specific responses [116]. From AS15 in 2015 to Pr20 in 2017, antibodies against PRAME have been well verified in animal models [117,118], and the new anti-PRAME monoclonal antibody developed in 2021 is more powerful and can recognize folded proteins on the surface of cell membranes [119]. In 2018, a report published in the journal Cell showed that CT45-derived HLA-I peptides can activate patient-derived cytotoxic T cells to kill and inhibit tumor progression [120]. Since KK-LC-1 belongs to the CTA family, we believe that it is possible to design antibodies against KK-LC-1 in the future.

In addition, an increasing number of studies have focused on a combination of targeted therapies against CTAs and immune checkpoint inhibitors[54,121,122]. In 2016, Lindy G Durrant’s team found that the long-term survival rate of mice was significantly improved after treatment with a combination of the NY-ESO-1 vaccine SCIB2 and anti-PD-1/CTLA-4, suggesting the feasibility of combination therapy [123]. In the future, SCIB2 can be used in the clinic. For patients with a low tumor mutation load, SCIB2 alone may be effective, but for patients with a high tumor mutation load, the effect of combined therapy may be more significant. In 2019, Mikio Oka’s team found that patients with non-small-cell lung cancer treated with NY-ESO-1 and XAGE1 monoclonal antibodies combined with anti-PD-1 had better efficacy and longer survival, and the antibody titer was positively correlated with the efficacy of anti-PD-1 therapy [124]. Therefore, we believe that NY-ESO-1 and XAGE1 antibodies are markers for predicting the efficacy of anti-PD-1 in patients with NSCLC and can be included in clinical trials of immune checkpoint inhibitors as stratification factors in the future. In 2020, Baoen Shan’s team found that although esophageal cancer expressed MAGE-A11, the expression level was not high, thus limiting the effectiveness of immunotherapy. MAGE-A11-derived CTLs can kill esophageal cancer cells expressing MAGE-A11 but have almost no killing effect on MAGE-A11-negative tumor cells [29]. At the same time, researchers found that PD-L1 can affect the antitumor function of CTLs. Therefore, they think a combination of DNA methyltransferase inhibitors and PDL1 inhibitors may be an effective approach. The results of cellular and animal experiments also confirmed that combination therapy could produce a specific cellular immune response to esophageal tumors, a promising strategy for clinical applications.

However, there is currently no literature reporting on a combination of KK-LC-1 and immune checkpoint inhibitors, and the simultaneous use of both may be a valuable method for clinical applications in the future.

## Concluding remarks

In summary, KK-LC-1 is a new target for immunotherapy and may become a valuable tumor-related marker in the future. Immunotherapy and combination therapy against KK-LC-1 may create new opportunities for cancer treatment. In the future, these new treatment strategies may result in new breakthroughs in cancer immunotherapy.
